# Optimizing the pharmacokinetics of an ^211^At-labeled RGD peptide with an albumin-binding moiety via the administration of an albumin-binding inhibitor

**DOI:** 10.1007/s00259-024-06695-w

**Published:** 2024-04-04

**Authors:** Hiroaki Echigo, Masayuki Munekane, Takeshi Fuchigami, Kohshin Washiyama, Kenji Mishiro, Hiroshi Wakabayashi, Kazuhiro Takahashi, Seigo Kinuya, Kazuma Ogawa

**Affiliations:** 1 Graduate School of Medical Sciences, Kanazawa University, Kakuma-Machi, Kanazawa, 920-1192 Ishikawa Japan; 2 Advanced Clinical Research Center, Fukushima Global Medical Science Center, Fukushima Medical University, 1 Hikarigaoka, Fukushima, 960-1295 Japan; 3 Institute for Frontier Science Initiative, Kanazawa University, Kakuma-Machi, Kanazawa, 920-1192 Ishikawa Japan; 4 Department of Nuclear Medicine, Kanazawa University Hospital, Kanazawa University, Takara-Machi 13-1, Kanazawa, 920-8641 Ishikawa Japan

**Keywords:** Radiotheranostics, Astatine-211, Targeted alpha therapy (TAT), RGD peptide, Albumin-binding moiety (ABM)

## Abstract

**Purpose:**

A probe for targeted alpha therapy (TAT) using the RGD peptide (Ga-DOTA-K([^211^At]APBA)-c(RGDfK) ([^211^At]**1**)) with albumin-binding moiety (ABM) was recently developed. [^211^At]**1** highly accumulated in tumors and significantly inhibited tumor growth in U-87 MG tumor-bearing mice. However, high [^211^At]**1** retention in blood may cause critical adverse events, such as hematotoxicity. Therefore, we attempted to accelerate the blood clearance of [^211^At]**1** by competitively inhibiting the binding of [^211^At]**1** to albumin to modulate the pharmacokinetics of the former.

**Methods:**

To evaluate the effects of albumin-binding inhibitors in normal mice, sodium 4-(4-iodophenyl)butanoate at 2, 5, or 10 molar equivalents of blood albumin was administered at 1-h postinjection of [^211^At]**1**. The biodistribution of [^211^At]**1**, SPECT/CT imaging of [^67^Ga]Ga-DOTA-K(IPBA)-c(RGDfK) ([^67^Ga]**2**), and the therapeutic effects of [^211^At]**1** were compared with or without IPBA administration in U-87 MG tumor-bearing mice.

**Results:**

Blood radioactivity of [^211^At]**1** was decreased in a dose-dependent manner with IPBA in normal mice. In U-87 MG tumor-bearing mice, the blood radioactivity and accumulation in nontarget tissues of [^211^At]**1** were decreased by IPBA. Meanwhile, tumor [^211^At]**1** accumulation was not changed at 3-h postinjection of IPBA. In SPECT/CT imaging of [^67^Ga]**2**, IPBA administration dramatically decreased radioactivity in nontarget tissues, and only tumor tissue was visualized. In therapeutic experiments, [^211^At]**1** with IPBA injected-group significantly inhibited tumor growth compared to the control group.

**Conclusion:**

IPBA administration (as an albumin-binding inhibitor) could modulate the pharmacokinetics and enhance the therapeutic effects of [^211^At]**1**.

**Supplementary Information:**

The online version contains supplementary material available at 10.1007/s00259-024-06695-w.

## Introduction

Radiotheranostics combines nuclear medicine imaging and radionuclide therapy [1–4]. Radionuclide therapy with α-emitters known as targeted alpha therapy (TAT) has attracted much attention due to its stellar therapeutic effects [5, 6]. Among various α-emitters, astatine-211 (^211^At) has become more popular because of its appropriate half-life (*t*_1/2_ = 7.2 h) and its producibility [7–9].

**Fig. 1:**
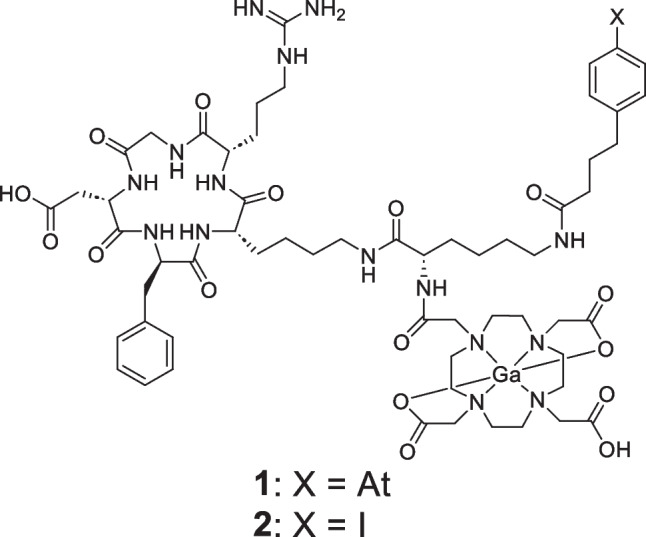
Chemical structures of Ga-DOTA-K(APBA)-c(RGDfK) (**1**) and Ga-DOTA-K(IPBA)-c(RGDfK) (**2**)

The therapeutic effects in nuclear medicine could be determined by the ratio of the radiation dose absorbed by the tumor to that absorbed by the tissue (the dose-limiting factor). Therefore, radionuclides for therapy should be highly accumulated and retained in tumor tissues to increase the absorbed dose, and their accumulation in nontarget tissues should be reduced. An albumin-binding moiety (ABM) is widely used to prolong the circulation half-lives of radiolabeled compounds [10]. As it is known that ABMs, such as 4-(4-iodophenyl)butyric acid (IPBA), have micromolar affinity and exhibit reversible binding to albumin, the ABM introduction can improve the pharmacokinetics of drugs [10–12]. Our research group previously developed ^211^At-labeled compounds for TAT with an Arg-Gly-Asp (RGD) peptide as a carrier to the tumor [13–15]. Recently, Ga-DOTA-K([^211^At]APBA)-c(RGDfK) ([^211^At]**1**) with 4-(4-astatophenyl)butyric acid (APBA) as ABM was developed to increase tumor accumulation (Fig. 1) [16]. [^211^At]**1** was developed using the concept of multiradionuclide radiotheranostics, which allows the use of a wide variety of radionuclides, including two radiolabeling sites for radiometal and radiohalogen. DOTA chelator was selected because it can coordinate with many types of radiometals stably, such as ^64^Cu, ^67/68^Ga, ^111^In, ^177^Lu, ^212/213^Bi, and ^225^Ac. In this study, ^67^Ga was used as an alternative radionuclide to ^68^Ga due to its longer half-life. Since APBA worked as a strong ABM, the tumor accumulation of [^211^At]**1** was significantly higher than that of other radiolabeled RGD peptides without ABM, and [^211^At]**1** manifested tumor growth inhibition in a dose-dependent manner. Therefore, the usefulness of [^211^At]**1** as the radionuclide therapy agent for TAT was revealed.

In the TAT of [^211^At]**1**, its prolonged blood clearance enhanced its tumor accumulation, potentially leading to critical adverse events, such as hematotoxicity [16]. Delayed blood clearance, which ensures high accumulation and prolonged tumor retention, is necessary to maximize the therapeutic effects of α-emitters with longer half-lives (such as ^225^Ac (*t*_1/2_ = 9.9 days)). However, as the half-life of ^211^At is not long, even if ^211^At is maintained in the tumor tissues for a long time, the absorbed dose does not increase. For ^211^At-labeled compounds, a high radioactivity accumulation ratio of the tumor over nontarget tissues within a short time after they are administered is essential to maximize their therapeutic effects. We supposed that the blood clearance of [^211^At]**1** was too slow for ideal ^211^At-labeled compounds for TAT. Therefore, to modulate the pharmacokinetics of [^211^At]**1**, we hypothesized that the blood clearance of [^211^At]**1** would be accelerated by the competitive inhibition of the [^211^At]**1** binding to albumin [17]. Namely, the concept is that an additional administration of an albumin-binding inhibitor after injection of [^211^At]**1** competitively inhibits the binding of [^211^At]**1** to albumin, thereby enhancing clearance.

Using ABM with lower affinity for albumin, such as 4-(4-tolylphenyl)butyric acid and 4-(4-chlorophenyl)butyric acid, than APBA could be one option to reduce blood retention instead of using an albumin-binding inhibitor [18]. However, the halogeno group of the benzene ring in ABM was used as a radiolabeling site in our compounds. Thus, we could not use 4-(4-tolylphenyl)butyric acid or 4-(4-chlorophenyl)butyric acid as ABM. Meanwhile, ABMs with different lengths of alkyl chains, such as 2-(4-iodophenyl)acetic acid, 3-(4-iodophenyl)propanoic acid, and 5-(4-iodophenyl)pentanoic acid, also have lower affinity for albumin [19, 20]. Although using them could be one option to reduce blood retention, tumor accumulation would also be reduced. Higher blood radioactivity could usually lead to higher tumor accumulation in the early stages after administration. Namely, using an albumin-binding inhibitor could give a higher absorbed dose to the tumor than using ABM with a lower affinity for albumin. Thus, we decided to use IPBA or APBA as ABMs with high affinity for albumin and attempted to accelerate blood clearance by administering an albumin-binding inhibitor. This study evaluated the efficacy of inhibitor administration in optimizing the pharmacokinetics of [^211^At]**1** and its therapeutic effect.

## Materials and methods

### General

^211^At was produced on CYPRIS MP-30 cyclotron (Sumitomo Heavy Industries, Ltd., Tokyo, Japan) in the Advanced Clinical Research Center at Fukushima Medical University [21]. [^125^I]Sodium iodide (629 GBq/mg) was purchased from PerkinElmer (Waltham, MA, USA). [^67^Ga]Ga-citrate was purchased from Nihon Medi-Physics Co., Ltd. (Tokyo, Japan), and converted [^67^Ga]GaCl_3_ by using Sep-Pak® Silica Plus Light Cartridge (Waters Co., Ltd., Milford, MA, USA) according to a previous report [22]. 4-(4-Iodophenyl)butyric acid was purchased from Ambeed (Arlington Heights, IL, USA). (2*RS*)-2-[4-(2-Methylpropyl)phenyl]propionic acid (ibuprofen) was purchased from Nacalai Tesque, Inc. (Kyoto, Japan). U-87 MG glioblastoma cells were purchased from DS Pharma Bio-medical (Osaka, Japan). Other reagents were of reagent grade and used as received.

### Preparation of radiolabeled compounds

Ga-DOTA-K([^211^At]APBA)-c(RGDfK) ([^211^At]**1**), [^67^Ga]Ga-DOTA-K(IPBA)-c(RGDfK) ([^67^Ga]**2**), and Ga-DOTA-K([^125^I]IPBA)-c(RGDfK) ([^125^I]**2**) were synthesized according to our previous report [16].

### Animals

Experiments with animals were conducted in strict accordance with the Guidelines for the Care and Use of Laboratory Animals of Kanazawa University. The experimental protocols were approved by the Committee on Animal Experimentation of Kanazawa University. The animals were housed with free access to food and water at 23 °C with a 12-h alternating light/dark schedule. Normal mice were used as 6-week-old male ddY mice (29–36 g, Japan SLC, Inc., Hamamatsu, Japan).

To prepare tumor-bearing mice, 5 × 10^6^ of U-87 MG cells were subcutaneously inoculated into the right shoulder of 4-week-old female BALB/c nude mice (13–15 g, Japan SLC, Inc.) as previously reported [23]. At approximately 10-day postinoculation of U-87 MG cells, they were used for each experiment after tumor size reached 0.3–0.5 cm^3^.

### Biodistribution experiments

To evaluate the effects of albumin-binding inhibitors in normal mice, [^211^At]**1** (37 kBq) and [^125^I]**2** (37 kBq) were intravenously coadministered. At 1-h postinjection of radiotracers, vehicle (saline), ibuprofen at 10 molar equivalent of blood albumin (2.5 mg, 11 µmol), or IPBA at 2 (680 µg, 2.2 µmol), 5 (1.7 mg, 5.5 µmol), or 10 (3.4 mg, 11 µmol) molar equivalent of blood albumin was administered. Ibuprofen and IPBA were administered as sodium salts. The total blood albumin (1.1 µmol) in normal mice was calculated from the albumin concentration (3.0 g/dL ≈ 455 µM) in blood, which was informed from Japan SLC, Inc., total blood volume (2.5 mL) as 8% of body weight, and the molecular weight of albumin as 66,000 g/mol. Mice were sacrificed at 4-h postinjection of radiotracers.

In the case of tumor-bearing mice, [^211^At]**1** (37 kBq) and [^125^I]**2** (37 kBq) were intravenously coadministered. At 1-h postinjection of [^211^At]**1** (37 kBq) and [^125^I]**2** (37 kBq), IPBA (1.9 mg, 6.1 µmol) was administered at 10 molar equivalent of blood albumin. Mice were sacrificed at 65-, 70-min, 2-, 4-, 12-, and 24-h postinjection of [^211^At]**1** and [^125^I]**2**. Tissues of interest were removed and weighed, and radioactivity counts of ^125^I and ^211^At were determined with an auto well gamma counter (ARC-7010; Hitachi Medical, Ltd., Tokyo, Japan) and corrected for background radiation [21]. To determine the radioactivity excreted from the body for 24 h, mice were housed in metabolic cages (Metabolica, Sugiyama-Gen Co., Ltd., Tokyo, Japan).

### Radiation dose estimation

For estimation of the radiation dose absorbed, the blood, bone, and muscle mass of mice were assumed to be 8%, 5%, and 48% of body weight, respectively [24]. The stomach was assumed to be distributed only in the wall, the heart, small intestine, and colon equally in the content and wall, and the colon was further assumed to be equally distributed in the left, right, and rectosigmoid. According to the International Commission on Radiological Protection, an equal distribution of radionuclide to trabecular and cortical bones was assumed [25]. The non-decay-corrected activity from each source organ was converted into a percentage of the injected dose. The area under each organ’s activity curve from time 0 to infinity was calculated by extrapolation of biodistribution data. To correct for the different ratios of organ to total body weights in mouse and in human, we used the following organ correction factor (CR):

$$\mathrm{CR}\;=\;{\left(\mathrm{organ}\;\mathrm{mass}/\mathrm{total}\;\mathrm{body}\;\mathrm{mass}\right)}_{\mathrm{human}}/{\left(\mathrm{organ}\;\mathrm{mass}/\mathrm{total}\;\mathrm{body}\;\mathrm{mass}\right)}_{\mathrm{mouse}}$$

Human organ weights and total body weights used data for adult males from previous reports [26]. Tumor volume was converted from mouse-to-human total body weight ratio. According to the values, the radiation doses were calculated for an adult male patient using MIRDcalc v1.21 software (Society of Nuclear Medicine and Molecular Imaging) [27]. In the case of without administrating IPBA, calculation was performed using the data of a biodistribution study in our previous study [16].

### SPECT/CT imaging and data reconstruction

SPECT/CT imaging of [^67^Ga]**2** with or without administration of IPBA in above-mentioned U-87 MG tumor-bearing mice was performed using a small animal SPECT system (VECTor/CT, MIlabs, Houten, the Netherlands). At 1-h postinjection of [^67^Ga]**2** (7.4 MBq), IPBA was administered at 10 molar equivalent of blood albumin. SPECT scanning was performed for 1 h from 3-h postinjection of [^67^Ga]**1**. The mice were sacrificed at 3-h postinjection because of long time acquisition of SPECT scanning.

Data were acquired in list mode, and photopeak windows were set after the acquisition. The energy windows of 80–110, 165–200, 265–320, and 350–410 keV were employed. Data were reconstructed using pixel-based order-subsets expectation maximization, with correction for attenuation on computed tomography, in 16 subsets and 6 iterations. Data were filtered with the 1-mm size Gaussian filter as the post filter. The voxel size was 0.8 × 0.8 × 0.8 mm. The obtained SPECT/CT images were analyzed using an image-processing application (AMIDE Imaging software, version 1.0.4, Slashdot Media, LCC., San Diego, CA, USA).

### Therapeutic experiments

U-87 MG tumor-bearing mice were divided into three groups, [^211^At]**1** (1.85 MBq) (*n* = 6) injected group, only [^211^At]**2** (1.85 MBq) and IPBA (*n* = 6) injected group, and vehicle and IPBA (*n* = 3) injected group as a control group. In IPBA injected group, it was administered at 10 molar equivalent of blood albumin at 1-h postinjection of [^211^At]**1** or vehicle. Tumor volume and body weight of mice were monitored 5 or 6 times weekly. Tumor size was measured with a slide caliper, and tumor volume was calculated using a formula: volume = 4/3 *π* (1/2 length × 1/2 width × 1/2 height). Tumor volume and body weight compared to the values on the day of treatment (relative tumor volume). The mice were euthanized humanely when body weight was less than 80% at baseline (day 0) or when the tumor weight reached more than 10% of body weight as the endpoint.

### Statistical evaluation

In biodistribution experiments, the difference between injected tracers with a double tracer method was determined by paired Student’s *t* test, and the difference among types and doses of inhibitors was determined by one-way analysis of variance (ANOVA) followed by Tukey–Kramer post hoc test. Therapeutic experiments were analyzed by unpaired Student’s *t* test.

## Results

### Preparation of radiolabeled compounds

The radiochemical yields of [^211^At]**1**, [^67^Ga]**2**, and [^125^I]**2** were 76%, 76%, and 83%, respectively. After HPLC purification, their radiochemical purities were > 97%. As HPLC purification completely separated the radiolabeled compounds from the precursors, the molar activity of [^211^At]**1**, [^125^I]**2**, and [^67^Ga]**2** was 1.6 × 10^4^, 8.1 × 10, and 1.5 × 10^3^ TBq/µmol, respectively.

### Biodistribution experiments

**Fig. 2:**
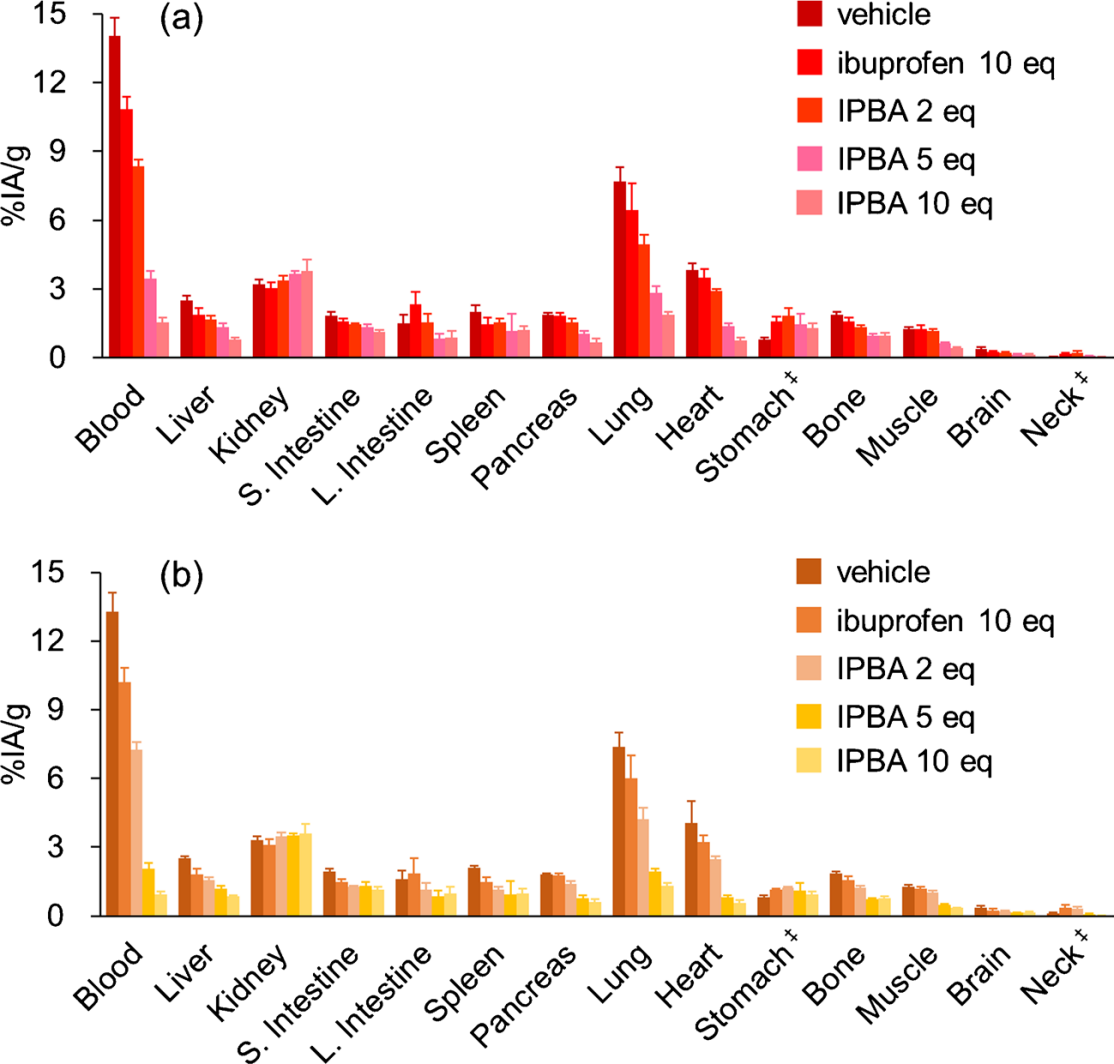
Biodistribution experiments. Biodistribution of radioactivity 4 h after administration of **a** [^211^At]**1** and **b** [^125^I]**2** in normal mice. Vehicle, ibuprofen, or IPBA were administrated at 1-h postinjection of [^211^At]**1** and [^125^I]**2**. ^‡^Expressed as % injected activity

Biodistribution experiments of [^211^At]**1** and [^125^I]**2** with the administration of IPBA or ibuprofen in normal mice to inhibit the blood albumin-binding were conducted, and the result of these experiments is presented in Fig. 2 and Table S1. Blood [^211^At]**1** radioactivity was decreased in a dose-dependent manner with IPBA. Due to the high blood retention of [^211^At]**1**, the radioactivity in nontarget tissues such as the lung and heart decreased dose-dependently with IPBA. Ibuprofen (as an inhibitor) did not significantly decrease the blood radioactivity of [^211^At]**1** much compared to IPBA.

**Fig. 3:**
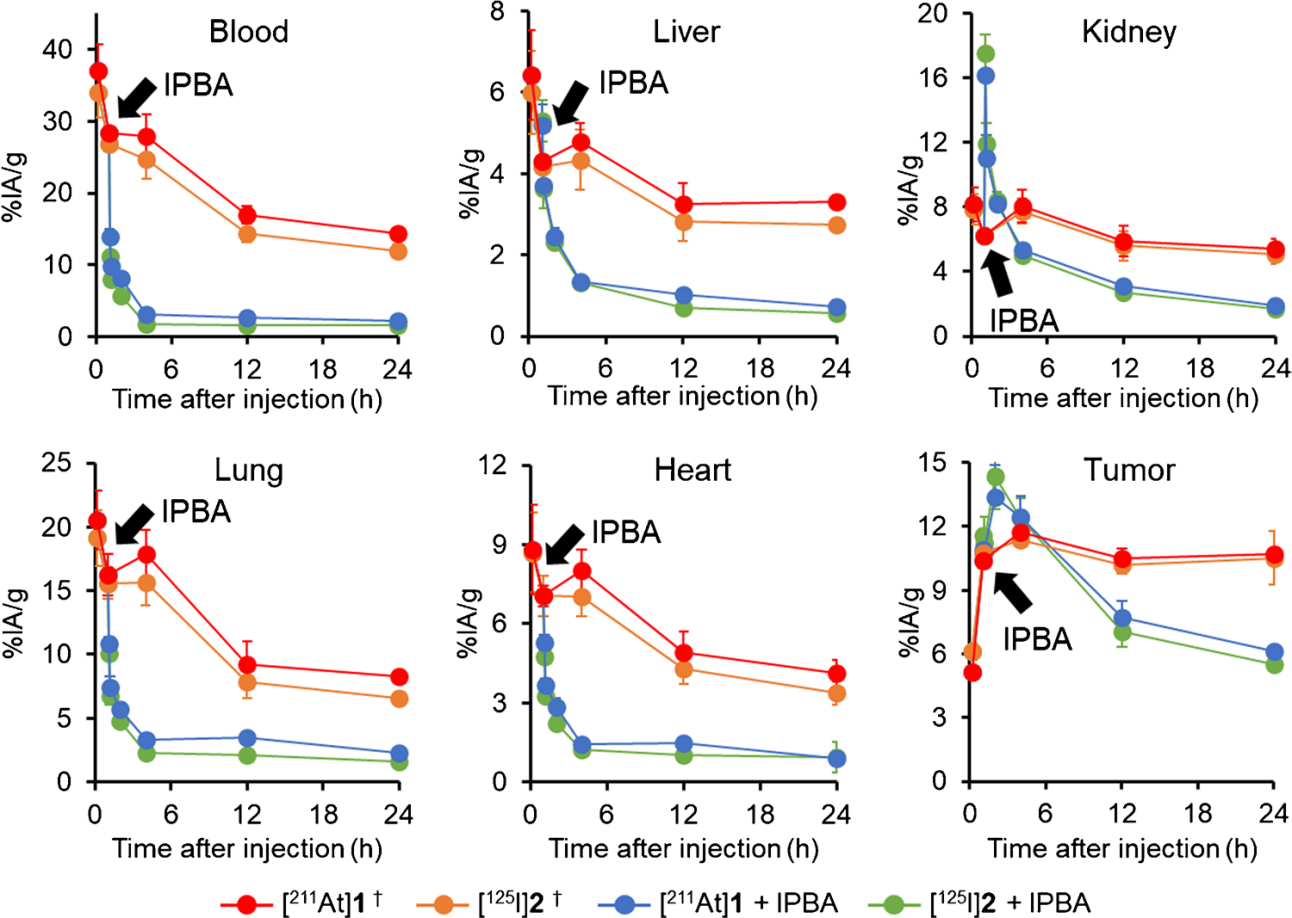
Biodistribution experiments. Comparison of biodistribution in U-87 MG tumor-bearing mice with or without administrating IPBA at 1-h postinjection of [^211^At]**1** and [^125^I]**2**. ^†^Data were originally published in Eur J Nucl Med Mol Imaging [16]

The biodistribution results with IPBA in U-87 MG tumor-bearing mice are shown in Fig. 3 and Table S3. Radioactivity in blood and nontarget tissues after the injection of [^211^At]**1** was decreased by administering IPBA. In contrast, kidney accumulation temporarily increased due to accelerated blood clearance by administrating IPBA; however, it decreased gradually. IPBA did not change tumor [^211^At]**1** accumulation at 4-h postinjection of [^211^At]**1**. At 24-h postinjection of [^211^At]**1**, tumor accumulation was significantly decreased by IPBA. However, the tumor accumulation (6.15 ± 0.20%IA/g) was significantly higher than that of [^67^Ga]Ga-DOTA-K-c(RGDfK) ([^67^Ga]**3**, Fig. S1) without ABM (1.84 ± 0.09%IA/g) [16]. Until 24-h postinjection, [^211^At]**1** excretion (urine: 38.98 ± 5.39, feces: 4.09 ± 1.69%IA) was significantly increased by the administration of IPBA compared to its nonadministration (urine: 10.94 ± 2.66, feces: 2.75 ± 0.40%IA).

[^125^I]**2** showed a similar biodistribution as [^211^At]**1** in both normal mice and U-87 MG tumor-bearing mice with or without IPBA (Figs. 2 and 3, Tables S1 and S3).

### Dosimetry

Table 1 shows the estimates of absorbed radiation doses of [^211^At]**1** with or without IPBA as an inhibitor and their ratios for values between normal tissues and tumor. The absorbed doses to tumors with or without IPBA administration were comparable. In contrast, the absorbed doses to nontarget tissues were decreased by IPBA administration because the IPBA administration immediately decreased the accumulation of radioactivity in nontarget tissues. As a result, the ratios of the absorbed dose in tumor tissue to that in nontarget tissues of [^211^At]**1** with IPBA were much higher than those of [^211^At]**1** without IPBA.

**Table 1:** Absorbed dose estimates of [^211^At]**1** and ratio between absorbed dose estimates for nontargeting tissues and tumor of [^211^At]**1** with IPBA as an inhibitor or not

Tissues	Absorbed dose estimates (mGy/MBq)		Tumor/normal tissue ratio	
	With IPBA	Without IPBA^†^	With IPBA	Without IPBA^†^
Bone marrow	0.042	0.132	20.380	6.500
Liver	0.109	0.319	7.872	2.690
Kidney	0.266	0.427	3.226	2.009
S. intestine	0.052	0.190	16.437	4.516
L. intestine	0.056	0.193	15.349	4.446
Spleen	0.117	0.354	7.333	2.424
Pancreas	0.087	0.246	9.873	3.488
Lung	0.203	0.976	4.227	0.879
Heart	0.080	0.381	10.712	2.252
Stomach	0.051	0.176	16.791	4.875
Muscle	0.300	0.557	2.860	1.540
Brain	0.017	0.218	51.071	3.936
Thyroid	0.032	0.111	26.729	7.730
Tumor	0.727	0.858		

^†^Each value was calculated from the data, which were originally published in Eur J Nucl Med Mol Imaging [16]

### SPECT/CT images

**Fig. 4:**
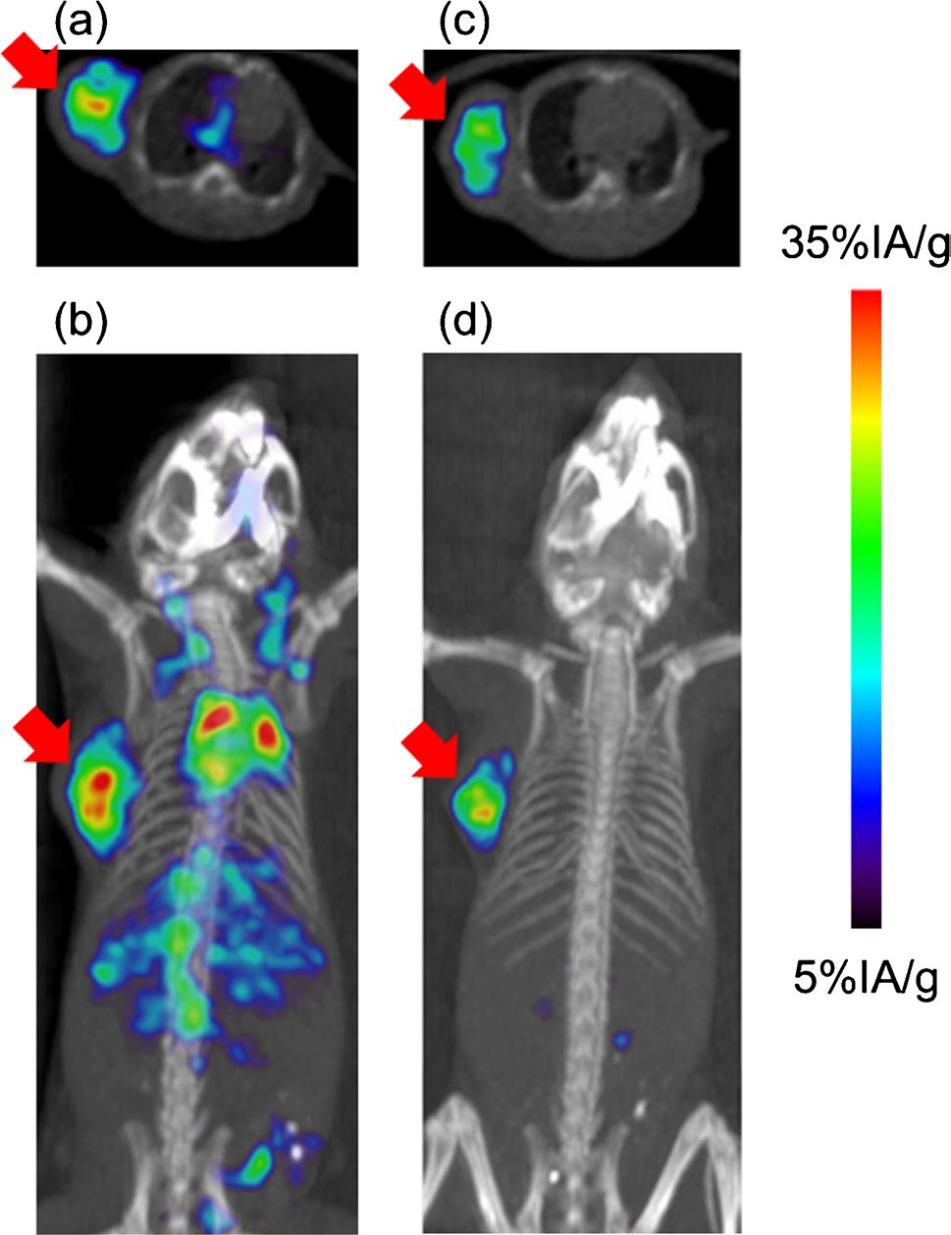
SPECT/CT images. **a** Axial SPECT/CT image and **b** coronal maximal intensity projection (MIP) SPECT/CT image without IPBA. **c** Axial SPECT/CT image and **d** coronal MIP SPECT/CT image with IPBA in U-87 MG tumor-bearing mice at 3-h postinjection of [^67^Ga]**2**. IPBA was administrated at 1-h post-injection of [^67^Ga]**2**. Arrows indicate the site where U-87 MG cells were inoculated

SPECT/CT images at 3-h postinjection of [^67^Ga]**2** with or without IPBA are presented in Fig. 4. [^67^Ga]**2** without IPBA images showed high accumulation in not only the tumor tissue but also normal tissues. IPBA administration dramatically decreased radioactivity in nontargeting tissues. Therefore, the only tumor tissue was visualized.

### Therapeutic experiments

**Fig. 5:**
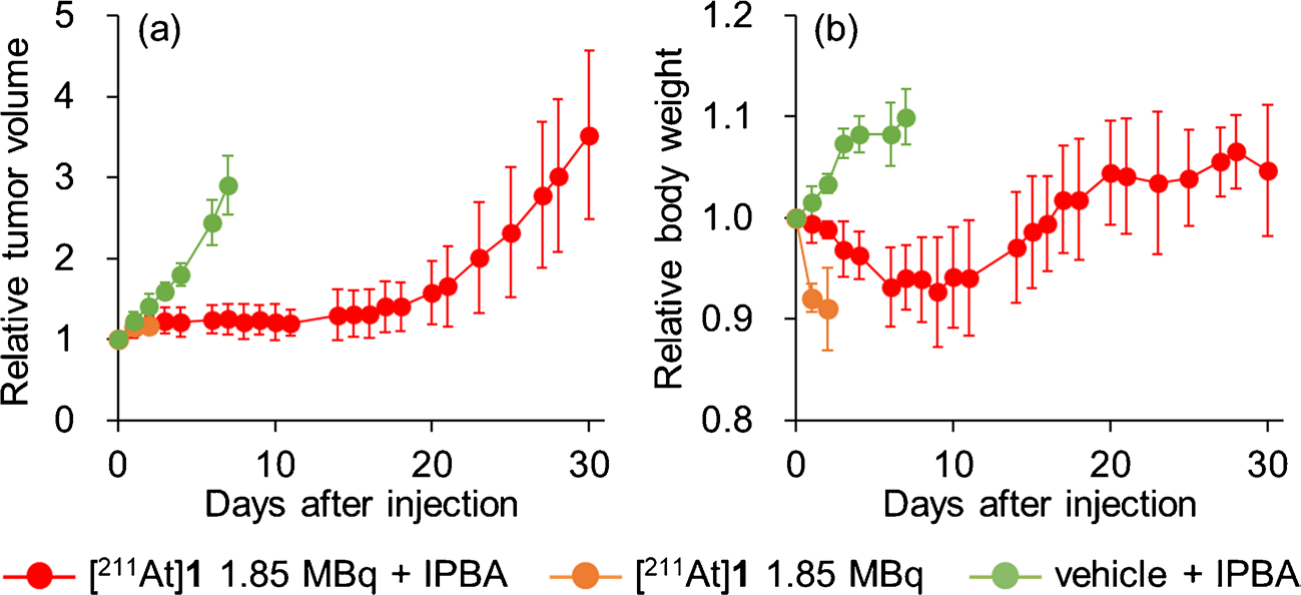
Therapeutic experiment. **a** Tumor volume and **b** body weight of U-87 MG tumor-bearing mice after administration of [^211^At]**1** 1.85 MBq with IPBA, [^211^At]**1** 1.85 MBq without IPBA, or vehicle with IPBA. Data are expressed as relative value to initial tumor volume (mean ± SD)

The differences in therapeutic effects and adverse events of [^211^At]**1** (1.85 MBq) with or without IPBA were evaluated. In the group where [^211^At]**1** was administered followed by IPBA, tumor growth was significantly more inhibited than it was in the control group. Conversely, in the group in which [^211^At]**1** was administered without IPBA, all mice died within 9 days with significant weight loss before their weight dropped below 80% as the endpoint. In the group in which [^211^At]**1** was administered followed by IPBA, although temporary weight loss was observed, the body weight loss was less than 10% at [^211^At]**1** administration, and the lost weight was recovered (Fig. 5).

## Discussion

IPBA was selected as an albumin-binding inhibitor because it has a common ABM (4-iodophenylpropyl group) with [^211^At]**1**. Thus, it is expected that IPBA binds to the same albumin-binding site as [^211^At]**1** with similar binding affinity and enables the competitive inhibition of [^211^At]**1**. Ibuprofen was also selected as an inhibitor because it is known to bind albumin-binding site II as well as IPBA [11]. Ibuprofen has been used in clinical practice for a long time. If ibuprofen is useful as an albumin-binding inhibitor, the strategy of using an inhibitor to cleave the albumin-binding of a radiopharmaceutical could be easily transferred to clinical practice.

Initially, we used normal mice to examine how much IPBA and ibuprofen dosage can accelerate probes’ blood clearance with ABM. IPBA or ibuprofen was administered at 1-h postinjection because the tumor accumulation of [^211^At]**1** and [^125^I]**2** had been plateau after 1-h postinjection [16]. As shown in Fig. 2, blood [^211^At]**1** radioactivity was decreased by IPBA administration. In addition, radioactivity in other tissues (such as the lung and heart) was significantly decreased due to a decrease in blood radioactivity. However, ibuprofen did not decrease blood [^211^At]**1** radioactivity as much as IPBA. This difference is explained by the fact that ibuprofen was used as a weaker ABM than IPBA [28, 29], and then, the binding affinity of ibuprofen for albumin could be lower than that of IPBA. Meanwhile, IPBA and [^211^At]**1** must have similar binding affinity for the same albumin-binding site due to their structural similarity. Ibuprofen and IPBA bind site II in the albumin-binding sites [11]. However, as ibuprofen also binds to several other binding sites in albumin [30], the binding sites of [^211^At]**1** and ibuprofen should not perfectly match.

Based on studies conducted on normal mice, ten equivalents of an inhibitor (IPBA) were administered in U-87 MG tumor-bearing mice because that number of equivalents of IPBA was considered necessary to sufficiently reduce blood radioactivity. In biodistribution studies conducted on U-87 MG tumor-bearing mice, blood retention and accumulation in nontarget tissues of [^211^At]**1** were significantly decreased by IPBA administration (as expected). The blood half-lives of [^211^At]**1** and [^125^I]**2** after IPBA administration were calculated from the results of biodistribution experiments in U-87 MG tumor-bearing mice to be 27.7 and 23.1 min, respectively. On the other hand, the blood half-lives of [^211^At]**1** and [^125^I]**2** without IPBA (after 1-h postinjection of [^211^At]**1** and [^125^I]**2**) were 50.7 and 44.1 h, respectively. These results indicate that IPBA administration considerably shortens the blood half-lives of [^211^At]**1** and [^125^I]**2**. The longer blood half-life of [^211^At]**1** than [^125^I]**2** is probably caused by the higher affinity of [^211^At]**1** for blood albumin than [^125^I]**2** [16]. Meanwhile, IPBA administration did not significantly alter tumor [^211^At]**1** accumulation at 4-h postinjection. This finding indicates that the administration of an inhibitor does not weaken the therapeutic effects of probes labeled with short half-life therapeutic radionuclides such as ^211^At.

In dosimetry calculations, the ratios of the absorbed dose in nontarget tissues to that in tumor tissues of [^211^At]**1** with IPBA were much higher than those of [^211^At]**1** without IPBA. In biodistribution experiments, the tumor accumulation of [^211^At]**1** at 24-h postinjection was significantly decreased by IPBA administration. However, the doses absorbed in tumors with or without IPBA administration were comparable. The biodistribution data are shown as decay-corrected values. On the other hand, the absorbed dose was calculated using the decayed values with a half-life of ^211^At. Therefore, the difference in tumor accumulation at 24-h postinjection has little impact on the absorbed doses. Meanwhile, inhibiting the binding of [^211^At]**1** to blood albumin reduced the dose absorbed by nontarget tissues while maintaining the dose absorbed by target tissues, as in previous studies [17]. Therefore, this method of inhibiting the binding of [^211^At]**1** to blood albumin can accelerate blood clearance, allowing [^211^At]**1** dose escalation and enhancing therapeutic efficacy without increasing adverse events.

When [^67/68^Ga]**2** or [^123^I]**2** is used for PET or SPECT imaging as diagnostic techniques for radiotheranostics, the high background due to their high blood retention may result in poor image contrast. Conversely, SPECT/CT imaging of [^67^Ga]**2** with IPBA decreased the accumulation in nontargeted tissues, resulting in the clear visualization of tumor tissue. These findings are consistent with the results of the biodistribution experiments. IPBA administration reduced background and clarified tumor tissue by reducing nontarget-tissue accumulation compared to its nonadministration. In this study, IPBA was administered 1 h after [^67^Ga]**2** injection, and SPECT/CT imaging was performed 2 h later (3-h postinjection of [^67^Ga]**2**). This time schedule is applicable for ^68^Ga-PET/CT imaging. Meanwhile, image contrast was greatly improved due to higher tumor accumulation and lower accumulation in nontarget tissues compared to our previous ^67^Ga-labeled RGD peptide [14].

In therapeutic experiments, [^211^At]**1** (1.85 MBq) followed by IPBA dramatically inhibited tumor growth without resulting in severe weight loss. However, the therapeutic effects of 925 kBq of [^211^At]**1** in our recent study [16] and 1.85 MBq of [^211^At]**1** with IPBA in this study seem to be not much different, even though the absorbed dose of 1.85 MBq of [^211^At]**1** with IPBA to tumor tissue (1.34 mGy) was higher than 925 kBq of [^211^At]**1** (0.74 mGy). Moreover, the tumor sizes were not reduced, although both cases of [^211^At]**1** therapy significantly inhibit tumor growth. It was reported that tumor accumulation and therapeutic effects of a radiolabeled compound in tumor-bearing mice were decreased with increasing tumor size [31] and that larger tumors were less radiosensitive than smaller tumors [32]. In this study, the relatively large tumor size (0.3–0.5 cm^3^) of the mice used in the therapeutic experiments may have limited the therapeutic effects.

In contrast, in the group where [^211^At]**1** (1.85 MBq) was administrated without IPBA, all mice died within 9 days before the body weight decreased less than 80% at baseline (day 0) as the endpoint. The short survival must have been caused by adverse events due to high blood retention and high accumulation in nontarget tissues. In our recent study [16], normal mice used for evaluating the toxicity of [^211^At]**1** showed no serious hematotoxicity and weight loss. U-87 MG tumor-bearing mice used for therapeutic experiments (370 and 925 kBq of [^211^At]**1**) showed a little weight loss, but no mice died. In this study, raising the dose of [^211^At]**1** to 1.85 MBq resulted in severe weight loss, and the mice died. Thus, 925 kBq is the maximum tolerated dose of [^211^At]**1** without IPBA. These findings indicate that the administration of IPBA after [^211^At]**1** administration results in high therapeutic effects while reducing the number of adverse events of [^211^At]**1**. This is because IPBA administration resulted in similar absorbed doses in tumors compared to its nonadministration but lower absorbed doses in nontarget tissues. In not only [^211^At]**1** but also other probes for radiotheranostics, the application of this strategy could be useful in the modulation of the pharmacokinetics of probes with ABM, which have problems of adverse events in therapy or poor contrast images in diagnosis due to persistent high blood retention.

This study aimed to demonstrate the concept that the binding of [^211^At]**1** to blood albumin can be inhibited by postadministration of an albumin-binding inhibitor, and the inhibition modulates pharmacokinetics and improves its radiation-absorbed dose. Optimizing the administration time, dose, and route while confirming and assuring the safety of an albumin-binding inhibitor will be necessary for future clinical studies. IPBA was used as an albumin-binding inhibitor in this study. However, it does not necessarily have to be IPBA if it shows similar effects. Investigating compounds among approved drugs that exhibit comparable or greater albumin-binding inhibition effects to IPBA may be one strategy for human application.

## Conclusion

This study revealed that the pharmacokinetics of [^211^At]**1** with ABM could be modulated by administering IPBA as an albumin-binding inhibitor. IPBA administration also suggested the possibility of radiotheranostics combining [^211^At]**1** and its corresponding imaging probe, such as [^67^Ga]**2**. This method may be applicable to the modulation of the pharmacokinetics of radiotheranostic probes with ABM, which induces adverse events due to high blood retention.

### Supplementary Information

Below is the link to the electronic supplementary material.

Supplementary file1 (DOCX 80.5 KB)  Preparation method of [^67^Ga]Ga-DOTA-K-c(RGDfK) ([^67^Ga]**3**), detailed biodistribution data in normal mice and tumor-bearing mice, and detailed therapeutic experiment data.

## Data Availability

Data are available from the corresponding author on reasonable request.
