# Supplementary material for: Optimizing the pharmacokinetics of an 211At-labeled RGD peptide with an albumin-binding moiety via the administration of an albumin-binding inhibitor
Source: Eur J Nucl Med Mol Imaging. 2024 Apr 4;51(9):2663–71. doi: 10.1007/s00259-024-06695-w (PMC11224111; doi:10.1007/s00259-024-06695-w)
Supplement: Supplementary file 1 — Supplementary file1 (DOCX 80.5 KB) Preparation method of [67Ga]Ga-DOTA-K-c(RGDfK) ([67Ga]3), detailed biodistribution data in normal mice and tumor-bearing mice, and detailed therapeutic experiment data. [file 259_2024_6695_MOESM1_ESM.docx]

**Supporting Information**

**Optimizing the pharmacokinetics of an ^211^At-labeled RGD peptide with an albumin-binding moiety via the administration of an albumin binding inhibitor**

Hiroaki Echigo^a^, Masayuki Munekane^a^, Takeshi Fuchigami^a^, Kohshin Washiyama^b^, Kenji Mishiro^c^, Hiroshi Wakabayashi^d^, Kazuhiro Takahashi^b^, Seigo Kinuya^d^,

Kazuma Ogawa*^, a, c^

^a^ *Graduate School of Medical Sciences, Kanazawa University, Kakuma-machi, Kanazawa, Ishikawa 920-1192, Japan;*

^b^ *Advanced Clinical Research Center, Fukushima Global Medical Science Center, Fukushima Medical University, 1 Hikarigaoka, Fukushima, 960-1295, Japan;*

^c^ *Institute for Frontier Science Initiative, Kanazawa University, Kakuma-machi, Kanazawa, Ishikawa 920-1192, Japan;*

^d^ *Department of Nuclear Medicine, Kanazawa University Hospital, Kanazawa University, Takara-machi 13-1, Kanazawa, Ishikawa 920-8641, Japan;*

***Corresponding Author**

Institute for Frontier Science Initiative; Kanazawa University; Kakuma-machi, Kanazawa 920-1192; Japan.

Telephone: +81-76-234-4460; Fax: +81-76-234-4460

E-mail: [kogawa@p.kanazawa-u.ac.jp](mailto:kogawa@p.kanazawa-u.ac.jp)

**Table of contents**

Preparation of [^67^Ga]Ga-DOTA-K-c(RGDfK) ([^67^Ga]**3**)S3

Figure S1 Chemical structure of Ga-DOTA-K-c(RGDfK) (**3**)S3

Biodistribution experiment of [^125^I]**2** and [^67^Ga]**3** in normal miceS3

Table S1 Biodistribution of [^211^At]**1** and [^125^I]**2** in normal mice S4

Table S2 Biodistribution of [^125^I]**2** and [^67^Ga]**3** in normal mice S6

Table S3 Biodistribution of [^211^At]**1** and [^125^I]**2** in U-87 MG tumor-bearing mice S8

Table S4 Relative tumor volume in therapeutic experiment S10

Table S5 Relative body weight in therapeutic experiment S10

Reference S11

**Preparation of [^67^Ga]Ga-DOTA-K-c(RGDfK) ([^67^Ga]3)**

[^67^Ga]Ga-DOTA-K-c(RGDfK) ([^67^Ga]**3**, Figure S1) were synthesized according to our previous report [1]. The radiochemical yield was 90%. After HPLC purification, its radiochemical purity was 99%. As HPLC purification completely separated the radiolabeled compound from the precursor, the molar activity of [^67^Ga]**3** was 1.5 × 10^3^ TBq/µmol.

**Figure S1.** Chemical structure of Ga-DOTA-K-c(RGDfK) (**3**)**.**

**Biodistribution experiment**

To evaluate the effects of inhibitors in normal mice, [^125^I]**2** (37 kBq) and [^67^Ga]**3** (37 kBq) were intravenously coadministered into 6 weeks male ddY mice (29-36 g, Japan SLC, Inc., Hamamatsu, Japan). At 1 h postinjection of radiotracers, sodium 4-(4-iodophenyl)butanoate (IPBA) at 2 (680 µg, 2.2 µmol) or 5 (1.7 mg, 5.5 µmol) molar equivalent of blood albumin was administered. Mice were sacrificed at 4 h post-injection of radiotracers. The result of this experiment is presented in Table S2. Blood radioactivity and accumulation in lung and heart of [^125^I]**2** were decreased dose-dependently with IPBA. In contrast, [^67^Ga]**3** without ABM did not change biodistribution by IPBA administration.

**Table S1.** Biodistribution of radioactivity 4 h after administration of [^211^At]**1** and [^125^I]**2** in normal mice. Ibuprofen or IPBA as an inhibitor was administered at 1 h postinjection of [^211^At]**1** and [^125^I]**2**.

| Tissues | vehicle | ibuprofen |  | IPBA | | |
| --- | --- | --- | --- | --- | --- | --- |
|  |  | 10 eq |  | 2 eq | 5 eq | 10 eq |
| [^211^At]**1** |  |  |  |  |  |  |
| Blood | 14.03 (0.79) | 10.82 (0.58) ^a^ |  | 8.33 (0.29) ^a, b^ | 3.46 (0.33) ^a, b, c^ | 1.56 (0.20) ^a, b, c, d^ |
| Liver | 2.51 (0.20) | 1.87 (0.31) ^a^ |  | 1.68 (0.15) ^a^ | 1.35 (0.16) ^a, b, c^ | 0.80 (0.07) ^a, b, c^ |
| Kidney | 3.19 (0.20) | 3.03 (0.24) |  | 3.36 (0.20) | 3.67 (0.10) ^b^ | 3.77 (0.50) ^b^ |
| S. intestine | 1.85 (0.16) | 1.58 (0.15) |  | 1.47 (0.05) | 1.35 (0.13) | 1.14 (0.08) ^a, b, d^ |
| L. intestine | 1.52 (0.35) | 2.32 (0.57) |  | 1.53 (0.39) | 0.84 (0.22) ^b^ | 0.88 (0.27) ^b^ |
| Spleen | 2.02 (0.26) | 1.47 (0.29) ^a^ |  | 1.54 (0.18) | 1.19 (0.73) ^a^ | 1.22 (0.17) ^a^ |
| Pancreas | 1.88 (0.10) | 1.85 (0.12) |  | 1.55 (0.15) ^a, b^ | 1.03 (0.15) ^a, b, c^ | 0.66 (0.17) ^a, b, c, d^ |
| Lung | 7.69 (0.63) | 6.44 (1.14) |  | 4.96 (0.40) ^a, b^ | 2.84 (0.27) ^a, b, c^ | 1.88 (0.12) ^a, b, c^ |
| Heart | 3.83 (0.30) | 3.49 (0.36) |  | 2.90 (0.09) ^a, b^ | 1.38 (0.13) ^a, b, c^ | 0.78 (0.10) ^a, b, c, d^ |
| Stomach^‡^ | 0.80 (0.10) | 1.57 (0.21) |  | 1.84 (0.32) | 1.45 (0.45) ^a, b^ | 1.30 (0.20) ^d^ |
| Bone | 1.88 (0.10) | 1.57 (0.18) ^a^ |  | 1.35 (0.06) ^a^ | 0.95 (0.08) ^a, b^ | 0.98 (0.12) ^a, b, c^ |
| Muscle | 1.26 (0.09) | 1.28 (0.15) |  | 1.16 (0.09) | 0.62 (0.04) ^a, b, c^ | 0.42 (0.03) ^a, b, c, d^ |
| Brain | 0.37 (0.11) | 0.26 (0.03) |  | 0.24 (0.04) ^a^ | 0.16 (0.01) ^a^ | 0.14 (0.02) ^a^ |
| Neck^‡^ | 0.05 (0.01) | 0.18 (0.03) ^a^ |  | 0.23 (0.07) ^a^ | 0.08 (0.02) ^a, b^ | 0.03 (0.02) ^b, c, d^ |
|  |  |  |  |  |  |  |
| [^125^I]**2** |  |  |  |  |  |  |
| Blood | 13.28 (0.80) ^***^ | 10.19 (0.64) ^***, a^ |  | 7.26 (0.34) ^***, a, b^ | 2.06 (0.24) ^***, a, b, c^ | 0.95 (0.12) ^**, a, b, c, d^ |
| Liver | 2.51 (0.07) | 1.81 (0.24) ^a^ |  | 1.58 (0.12) ^a^ | 1.20 (0.12) ^*, a, b, c^ | 0.84 (0.06) ^a, b, c^ |
| Kidney | 3.32 (0.15) | 3.10 (0.24) |  | 3.46 (0.17) ^*^ | 3.52 (0.08) ^*, b^ | 3.60 (0.43) |
| S. intestine | 1.94 (0.14) ^**^ | 1.49 (0.12) ^*^ |  | 1.28 (0.03) ^**, a^ | 1.33 (0.14) | 1.17 (0.13) ^a^ |
| L. intestine | 1.59 (0.38) | 1.87 (0.66) ^*^ |  | 1.14 (0.31) ^**^ | 0.87 (0.25) | 0.98 (0.31) ^*, b^ |
| Spleen | 2.11 (0.08) | 1.49 (0.18) ^a^ |  | 1.15 (0.11) ^*, a, b^ | 0.93 (0.58) ^a, b^ | 1.00 (0.19) ^**, a, b^ |
| Pancreas | 1.81 (0.05) | 1.78 (0.09) |  | 1.41 (0.10) ^*, a, b^ | 0.79 (0.10) ^**, a, b, c^ | 0.60 (0.15) ^a, b, c, d^ |
| Lung | 7.38 (0.61) ^*^ | 6.02 (0.99) ^*, a^ |  | 4.22 (0.49) ^***, a, b^ | 1.92 (0.16) ^**, a, b, c^ | 1.33 (0.09) ^***, a, b, c^ |
| Heart | 4.05 (0.98) | 3.24 (0.29) ^*^ |  | 2.49 (0.13) ^**, a^ | 0.83 (0.09) ^**, a, b, c^ | 0.58 (0.10) ^***, a, b, c^ |
| Stomach^‡^ | 0.80 (0.12) | 1.16 (0.03) ^*^ |  | 1.21 (0.07) ^*^ | 1.11 (0.34) ^**, a^ | 0.94 (0.12) ^**^ |
| Bone | 1.84 (0.12) | 1.56 (0.16) ^a^ |  | 1.22 (0.11) ^a, b^ | 0.73 (0.06) ^**, a, b, c^ | 0.77 (0.07) ^**, a, b, c^ |
| Muscle | 1.28 (0.06) | 1.19 (0.07) |  | 1.02 (0.07) ^*, a, b^ | 0.47 (0.04) ^**, a, b, c^ | 0.34 (0.34) ^*, a, b, c, d^ |
| Brain | 0.38 (0.09) | 0.25 (0.05) ^a^ |  | 0.20 (0.03) ^*, a^ | 0.15 (0.02) ^a^ | 0.15 (0.03) ^a^ |
| Neck^‡^ | 0.13 (0.03) ^*^ | 0.36 (0.12) ^*, a^ |  | 0.32 (0.10) | 0.07 (0.02) ^a^ | 0.03 (0.02) ^b, c, d^ |

Expressed as % injected activity per gram.

Each value represents the mean (SD) for four animals.

^‡^ Expressed as % injected activity.

Significance between [^211^At]**1** and [^125^I]**2** was determined by paired Student’s *t* test. Significance among vehicle, ibuprofen, and each dose of IPBA was determined by one-way analysis of variance (ANOVA) followed by Tukey-Kramer post hoc test.

**p* < 0.05, ***p* < 0.01, ****p* < 0.001 vs [^211^At]**1**.

^a^*p* < 0.05 vs vehicle, ^b^*p* < 0.05 vs ibuprofen (10 eq), ^c^*p* < 0.05 vs IPBA (2 eq), ^d^*p* < 0.05 vs IPBA (5 eq).

**Table S2.** Biodistribution of radioactivity 4 h after administration of [^125^I]**2** and [^67^Ga]**3** in normal mice. IPBA as an inhibitor was administered at 1 h postinjection of [^125^I]**2** and [^67^Ga]**3**.

| Tissues | without IPBA^†^ | 2 eq | 5 eq |
| --- | --- | --- | --- |
| [^125^I]**2** |  |  |  |
| Blood | 11.80 (0.75) | 7.32 (0.73) ^a^ | 3.53 (0.77) ^a, b^ |
| Liver | 1.96 (0.18) | 1.54 (0.09) | 1.33 (0.31) |
| Kidney | 3.70 (0.65) | 4.13 (0.45) | 4.79 (1.04) ^a^ |
| S. intestine | 1.58 (0.31) | 1.19 (0.09) | 1.39 (0.32) |
| L. intestine | 1.20 (0.25) | 0.94 (0.07) | 1.11 (0.23) |
| Spleen | 1.61 (0.12) | 1.29 (0.23) | 1.40 (0.28) |
| Pancreas | 2.05 (0.25) | 1.42 (0.03) ^a^ | 1.17 (0.22) ^a^ |
| Lung | 5.60 (0.52) | 4.32 (0.41) ^a^ | 3.10 (0.74) ^a^ |
| Heart | 3.81 (0.18) | 2.36 (0.10) ^a^ | 1.52 (0.66) ^a, b^ |
| Stomach^‡^ | 0.87 (0.11) | 1.08 (0.18) | 1.35 (0.34) |
| Bone | 1.50 (0.26) | 1.27 (0.17) | 1.18 (0.19) |
| Muscle | 1.32 (0.16) | 1.02 (0.09) | 0.64 (0.12) ^a, b^ |
| Brain | 0.29 (0.03) | 0.23 (0.03) | 0.19 (0.03) |
| Neck^‡^ | 0.09 (0.02) | 0.05 (0.01) | 0.06 (0.00) |
|  |  |  |  |
| [^67^Ga]**3** |  |  |  |
| Blood | 0.09 (0.05) ^***^ | 0.03 (0.01) ^***, a^ | 0.03 (0.00) ^*, a^ |
| Liver | 1.15 (0.07) ^**^ | 1.18 (0.14) ^***^ | 1.54 (0.48) |
| Kidney | 6.61 (0.81) ^*^ | 6.67 (1.04) ^*^ | 8.04 (0.06) |
| S. intestine | 1.16 (0.15) ^*^ | 1.05 (0.22) | 1.76 (0.40) ^*, b^ |
| L. intestine | 1.88 (0.65) | 3.07 (2.64) | 4.88 (1.50) ^a^ |
| Spleen | 0.64 (0.12) ^***^ | 0.64 (0.16) ^***^ | 0.90 (0.28) ^*^ |
| Pancreas | 0.40 (0.03) ^***^ | 0.33 (0.01) ^***^ | 0.47 (0.13) ^**^ |
| Lung | 0.50 (0.04) ^***^ | 0.49 (0.05) ^***, a^ | 0.55 (0.11) ^*, a^ |
| Heart | 0.26 (0.04) ^***^ | 0.23 (0.03) ^***, a^ | 0.29 (0.07) |
| Stomach^‡^ | 0.44 (0.04) ^*^ | 0.48 (0.09) ^***^ | 0.59 (0.11) ^*^ |
| Bone | 0.83 (0.05) ^*^ | 0.55 (0.08) ^**, a^ | 0.70 (0.12) ^**^ |
| Muscle | 0.19 (0.02) ^***^ | 0.16 (0.02) ^***, a^ | 0.23 (0.04) ^*, b^ |
| Brain | 0.05 (0.00) ^***^ | 0.04 (0.01) ^**^ | 0.05 (0.02) ^**^ |
| Neck^‡^ | 0.02 (0.00) ^**^ | 0.01 (0.00) ^**^ | 0.01 (0.00) ^**^ |

Expressed as % injected activity per gram.

Each value represents the mean (SD) for three or four animals.

^†^Data were originally published in *Eur J Nucl Med Mol Imaging* [1].

^‡^ Expressed as % injected activity.

Significance between [^125^I]**2** and [^67^Ga]**3** was determined by paired Student’s *t* test. Significance between without inhibitor and each dose of IPBA was determined by ANOVA followed by Tukey-Kramer post hoc test.

**p* < 0.05, ***p* < 0.01, ****p* < 0.001 vs [^125^I]**2.**  ^a^*p* < 0.05 vs without inhibitor, ^b^*p* < 0.05 vs IPBA (2 eq).

**Table S3.** Biodistribution of radioactivity 65, 70 min, 2, 4, 12, and 24 h after administration of [^211^At]**1** and [^125^I]**2** in U-87 MG tumor-bearing mice. IPBA as an inhibitor was administered at 1 h postinjection of [^211^At]**1** and [^125^I]**2**.

| Tissues | 65 min | 70 min | 2 h | 4 h | 12 h | 24 h |
| --- | --- | --- | --- | --- | --- | --- |
| [^211^At]**1** |  |  |  |  |  |  |
| Blood | 13.96 (0.18) | 9.85 (0.55) | 8.06 (0.39) | 3.04 (0.26) | 2.71 (0.21) | 2.21 (0.38) |
| Liver | 5.20 (0.50) | 3.72 (0.52) | 2.45 (0.21) | 1.34 (0.05) | 1.04 (0.12) | 0.73 (0.17) |
| Kidney | 16.15 (1.45) | 11.02 (1.44) | 8.22 (0.51) | 5.32 (0.35) | 3.12 (0.14) | 1.91 (0.38) |
| S. intestine | 4.69 (0.26) | 3.61 (0.36) | 2.42 (0.70) | 3.03 (0.65) | 1.01 (0.14) | 0.46 (0.22) |
| L. intestine | 3.05 (0.10) | 2.25 (0.27) | 2.35 (0.97) | 4.03 (1.33) | 0.89 (0.06) | 0.41 (0.24) |
| Spleen | 4.06 (0.21) | 2.77 (0.39) | 2.34 (0.18) | 2.18 (0.13) | 1.41 (0.12) | 1.22 (0.27) |
| Pancreas | 2.82 (0.16) | 1.95 (0.14) | 1.64 (0.14) | 0.93 (0.26) | 0.92 (0.08) | 0.41 (0.12) |
| Lung | 10.81 (0.60) | 7.40 (0.85) | 5.67 (0.59) | 3.34 (0.11) | 3.50 (0.28) | 2.31 (0.34) |
| Heart | 5.28 (0.29) | 3.66 (0.27) | 2.86 (0.31) | 1.44 (0.10) | 1.49 (0.11) | 0.90 (0.31) |
| Stomach^‡^ | 1.12 (0.28) | 0.78 (0.17) | 0.81 (0.05) | 0.75 (0.01) | 0.72 (0.09) | 0.61 (0.18) |
| Bone | 4.07 (0.26) | 2.22 (0.60) | 3.04 (0.30) | 1.60 (0.14) | 1.10 (0.24) | 0.64 (0.16) |
| Muscle | 2.80 (0.39) | 1.60 (0.16) | 1.96 (0.68) | 0.61 (0.01) | 0.54 (0.02) | 0.32 (0.07) |
| Brain | 0.36 (0.00) | 0.26 (0.02) | 0.27 (0.68) | 0.18 (0.02) | 0.12 (0.01) | 0.26 (0.31) |
| Neck^‡^ | 0.04 (0.02) | 0.05 (0.00) | 0.04 (0.02) | 0.02 (0.00) | 0.09 (0.03) | 0.04 (0.01) |
| Tumor | 10.92 (0.34) | 10.79 (1.09) | 13.38 (1.48) | 12.48 (0.95) | 7.73 (0.76) | 6.15 (0.20) |
| Urine^‡^ |  |  |  |  |  | 38.98 (5.39) |
| Feces^‡^ |  |  |  |  |  | 4.09 (1.69) |
|  |  |  |  |  |  |  |
| [^125^I]**2** |  |  |  |  |  |  |
| Blood | 11.19 (0.16) ^**^ | 7.91 (0.45) ^***^ | 5.69 (0.44) ^***^ | 1.71 (0.12) ^**^ | 1.65 (0.20) ^***^ | 1.55 (0.17) ^*^ |
| Liver | 5.29 (0.52) ^*^ | 3.64 (0.49) ^*^ | 2.31 (0.17) ^*^ | 1.32 (0.06) | 0.70 (0.08) ^***^ | 0.56 (0.10) ^*^ |
| Kidney | 17.52 (1.13) ^*^ | 11.87 (1.35) ^***^ | 8.39 (0.51) | 5.00 (0.36) ^**^ | 2.71 (0.17) ^***^ | 1.67 (0.24) |
| S. intestine | 4.93 (0.21) ^*^ | 3.77 (0.37) ^**^ | 2.41 (0.71) | 3.09 (0.79) | 0.68 (0.10) ^***^ | 0.26 (0.11) |
| L. intestine | 3.10 (0.11) | 2.29 (0.24) | 2.48 (1.12) | 4.61 (1.33) ^**^ | 0.75 (0.05) ^***^ | 0.38 (0.22) |
| Spleen | 3.95 (0.26) | 2.83 (0.36) | 2.33 (0.14) | 1.83 (0.10) ^*^ | 1.03 (0.05) ^**^ | 0.72 (0.16) ^*^ |
| Pancreas | 2.66 (0.10) | 1.82 (0.14) ^*^ | 1.68 (0.36) | 0.72 (0.15) | 0.50 (0.02) ^**^ | 0.27 (0.05) |
| Lung | 10.10 (0.52) ^**^ | 6.75 (0.74) ^**^ | 4.74 (0.12) ^*^ | 2.31 (0.06) ^***^ | 2.07 (0.11) ^***^ | 1.58 (0.42) |
| Heart | 4.77 (0.10) | 3.25 (0.25) ^**^ | 2.24 (0.23) ^**^ | 1.22 (0.12) | 1.02 (0.07) ^**^ | 0.92 (0.58) |
| Stomach^‡^ | 1.33 (0.31) | 0.78 (0.15) | 0.77 (0.08) | 0.65 (0.09) | 0.39 (0.02) ^**^ | 0.20 (0.04) ^*^ |
| Bone | 4.94 (1.52) | 2.30 (0.36) | 2.73 (0.47) | 1.46 (0.16) | 0.72 (0.09) ^*^ | 0.48 (0.04) |
| Muscle | 2.62 (0.24) | 1.53 (0.18) | 1.73 (0.60) ^*^ | 0.52 (0.05) ^*^ | 0.37 (0.01) ^***^ | 0.26 (0.02) |
| Brain | 0.31 (0.02) | 0.24 (0.04) | 0.24 (0.02) | 0.18 (0.01) | 0.07 (0.01) ^**^ | 0.05 (0.01) |
| Neck^‡^ | 0.05 (0.01) ^*^ | 0.06 (0.01) | 0.08 (0.04) | 0.05 (0.04) | 0.06 (0.02) ^*^ | 0.03 (0.00) |
| Tumor | 11.58 (0.32) ^*^ | 11.24 (1.19) ^**^ | 14.36 (1.54) ^**^ | 12.42 (0.89) | 7.08 (0.74) ^**^ | 5.51 (0.05) ^*^ |
| Urine^‡^ |  |  |  |  |  | 56.09 (4.93) ^**^ |
| Feces^‡^ |  |  |  |  |  | 8.40 (3.14) |

Expressed as % injected activity per gram.

Each value represents the mean (SD) for three animals.

^‡^ Expressed as % injected activity.

Significance was determined by paired Student’s *t* test.

**p* < 0.05, ***p* < 0.01, ****p* < 0.001 vs [^211^At]**1**.

**Table S4.** Relative tumor volume of U-87 MG tumor-bearing mice after administration of [^211^At]**1** (1.85 MBq) or vehicle followed by IPBA. Data are expressed as relative value to initial tumor volume (mean ± SD).

| Days after injection | [^211^At]**1** (1.85 MBq) with IPBA | Vehicle with IPBA |
| --- | --- | --- |
| 0 | 1.00 (0.00) | 1.00 (0.00) |
| 1 | 1.14 (0.11) | 1.23 (0.11) |
| 2 | 1.19 (0.09) | 1.41 (0.16) ^*^ |
| 3 | 1.23 (0.15) | 1.59 (0.11) ^**^ |
| 4 | 1.21 (0.18) | 1.80 (0.14) ^**^ |
| 6 | 1.24 (0.17) | 2.45 (0.28) ^***^ |
| 7 | 1.25 (0.19) | 2.91 (0.36) ^***^ |

Data are expressed as relative value to initial tumor volume mean (SD) for three or six animals.

Significance was determined by unpaired Student’s *t* test.

**p* < 0.05, ***p* < 0.01, ****p* < 0.001 vs [^211^At]**1** with IPBA.

**Table S5.** Relative body weight of U-87 MG tumor-bearing mice after administration of [^211^At]**1** (1.85 MBq) or vehicle followed by IPBA. Data are expressed as relative value to initial body weight (mean ± SD).

| Days after injection | [^211^At]**1** (1.85 MBq) with IPBA | Vehicle with IPBA |
| --- | --- | --- |
| 0 | 1.00 (0.00) | 1.00 (0.00) |
| 1 | 0.99 (0.02) | 1.02 (0.02) |
| 2 | 0.99 (0.01) | 1.03 (0.01) ^***^ |
| 3 | 0.97 (0.03) | 1.07 (0.02) ^***^ |
| 4 | 0.96 (0.02) | 1.08 (0.02) ^***^ |
| 6 | 0.93 (0.04) | 1.08 (0.03) ^***^ |
| 7 | 0.94 (0.03) | 1.10 (0.03) ^***^ |

Data are expressed as relative value to initial tumor volume mean (SD) for three or six animals.

Significance was determined by unpaired Student’s *t* test.

****p* < 0.001 vs [^211^At]**1** with IPBA.

**Reference**

1. Echigo H, Mishiro K, Munekane M, Fuchigami T, Washiyama K, Takahashi K, et al. Development of probes for radiotheranostics with albumin binding moiety to increase the therapeutic effects of astatine-211 (^211^At). Eur J Nucl Med Mol Imaging. 2024;51:412-21. doi:10.1007/s00259-023-06457-0.
